# Biomimetic Alginate/Gelatin Cross-Linked Hydrogels Supplemented with Polyphosphate for Wound Healing Applications

**DOI:** 10.3390/molecules25215210

**Published:** 2020-11-09

**Authors:** Shunfeng Wang, Xiaohong Wang, Meik Neufurth, Emad Tolba, Hadrian Schepler, Shichu Xiao, Heinz C. Schröder, Werner E. G. Müller

**Affiliations:** 1 ERC Advanced Investigator Grant Research Group at the Institute for Physiological Chemistry, University Medical Center of the Johannes Gutenberg University, Duesbergweg 6, D-55128 Mainz, Germany; shunwang@uni-mainz.de (S.W.); mneufurt@uni-mainz.de (M.N.); emad_nrc@yahoo.com (E.T.); hschroed@uni-mainz.de (H.C.S.); 2 Department of Dermatology, University Clinic Mainz, Langenbeckstr. 1, D-55131 Mainz, Germany; Hadrian.Schepler@unimedizin-mainz.de; 3 Shanghai Changhai Hospital, No. 168 Changhai Road, Yangpu District, Shanghai 200000, China; huangzhuoxiao@hotmail.com

**Keywords:** inorganic polyphosphate, coacervate, nanoparticles, alginate, periodate oxidation, gelatin, ionic cross-linking, zinc ions, cell migration, human epidermal keratinocytes

## Abstract

In the present study, the fabrication of a biomimetic wound dressing that mimics the extracellular matrix, consisting of a hydrogel matrix composed of non-oxidized and periodate-oxidized marine alginate, was prepared to which gelatin was bound via Schiff base formation. Into this alginate/oxidized-alginate-gelatin hydrogel, polyP was stably but reversibly integrated by ionic cross-linking with Zn^2+^ ions. Thereby, a soft hybrid material is obtained, consisting of a more rigid alginate scaffold and porous structures formed by the oxidized-alginate-gelatin hydrogel with ionically cross-linked polyP. Two forms of the Zn-polyP-containing matrices were obtained based on the property of polyP to form, at neutral pH, a coacervate—the physiologically active form of the polymer. At alkaline conditions (pH 10), it will form nanoparticles, acting as a depot that is converted at pH 7 into the coacervate phase. Both polyP-containing hydrogels were biologically active and significantly enhanced cell growth/viability and attachment/spreading of human epidermal keratinocytes compared to control hydrogels without any adverse effect on reconstructed human epidermis samples in an in vitro skin irritation test system. From these data, we conclude that polyP-containing alginate/oxidized-alginate-gelatin hydrogels may provide a suitable regeneratively active matrix for wound healing for potential in vivo applications.

## 1. Introduction

**Figure 1:**
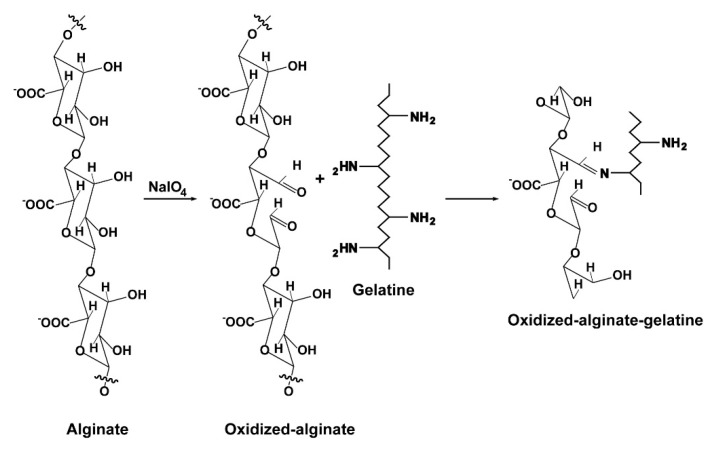
Oxidation of sodium alginate by periodate (NaIO_4_) and cross-linking with gelatine.

The majority of cells in animal/human tissues is embedded into an extracellular matrix (ECM), which consists of an organized meshwork of proteins and polysaccharides synthesized by the cells. The ECM is a hydrogel, a highly water swollen polymer network [1] with a mechanical behavior allowing cell migration, proliferation, and differentiation. For application in tissue engineering, a biomimetically fabricated hydrogel matrix must be composed that mimics the ECM of the native tissue with respect to the physical and physico-chemical/mechanical properties and demands. Among the most intensively studied hydrogels is sodium alginate, which is extracted from marine brown algae and structured as a block copolymer that comprises 1,4-linked *β*-d-mannuronic acid (M) with ^4^C_1_ ring conformation and *α*-L-guluronic acid (G) with ^1^C_4_ conformation (reviewed in: [2]). If exposed to divalent cations, like Ca^2+^, alginate forms a relatively stiff hydrogel that is not optimal for cells to migrate [3]. To overcome potential disadvantages of alginate an alginate derivative, oxidized alginate (also termed alginate dialdehyde) has been developed (see: [2]). By oxidation and formation of aldehyde groups within the original alginate chain, the polymer gains additional functionalities such as the ability of the polymer to covalently bind to amine-containing biomaterials such as proteins through Schiff base formation [4]; Figure 1. One prominent example is the cross-linking of oxidized alginate with gelatin via the ε-amino groups of lysine and hydroxylysine. Integration of gelatin transforms the alginate-oxidized-alginate matrix in a hydrogel with improved cell-material interaction and higher biocompatibility combined with faster biodegradation but still allowing the fabrication of three-dimensional (3D) printed matrices.

However, the disadvantage of alginate-oxidized alginate hydrogels is the lack of components that interact with the embedded cells in a morphogenetic manner and/or as contributor or source of metabolic energy. In recent years, the physiological inorganic polymer polyphosphate (polyP) moved into the focus of tissue engineering. This polymer, which is abundant in all metazoan cells, especially in blood platelets [5], combines these two requirements. It is morphogenetically active by inducing basic genes whose proteins are required for the construction of a substituting ECM, like collagen [6,7]. This beneficial property is added to the property of polyP to act as a generator of metabolic energy [8]. In this process of extracellular ATP generation from polyP two enzymes are involved, first alkaline phosphatase and second adenylate kinase. The alkaline phosphatase hydrolytically cleaves the energy-rich phosphoanhydride bond, during which the released energy is conserved in ADP; then ADP is enzymatically converted into ATP and AMP by the adenylate kinase [9].

In the present study, we intend to use an alginate/oxidized-alginate-gelatin matrix as a hydrogel applicable for wound healing. A suitable wound dressing should allow both maintenance and, at the same time, moisture exchange and parallel gas exchange. In addition, the material should absorb excess wound exudate and finally protect the wound from microbial infection that adversely affects the lesion from the environment.

Already in 2005, Balakrishnan et al. [10] proposed and applied alginate/oxidized-alginate together with gelatin as a hydrogel for application in the field of wound healing, after self-cross-linking of the components. In the present study, we followed this strategy and integrated polyP into the alginate/oxidized-alginate-gelatin matrix. The interaction of polyP with the hydrogel is based on ionic interactions. In addition, we used as a counterion Zn^2+^ (zinc ion) since this ion is critical for initiation of angiogenesis during the wound healing process [11]. Zn^2+^ ions are essential cofactors for zinc-dependent endopeptidases, acting during wound healing, like matrix metallopeptidase-9, an enzyme that promotes cell growth through hydrolysis of collagen molecules. Zinc is readily ionically linked to polyP and released again from the polymer after its enzymatic cleavage by alkaline phosphatase.

In the present study we describe the techniques to fabricate oxidized alginate from alginate and to link this hydrogel with the biologically active polyP in the presence of Zn^2+^. After suitable chemical and physico-chemical characterization the formed hydrogel was tested for biological activity (growth/viability) and also for the property to allow the cells to migrate. Based on the data presented further application of the hydrogel for in vivo studies is recommended.

## 2. Results

### 2.1. Preparation of Zn-polyP-coacervate and Zn-polyP-nanoparticles

**Figure 2:**
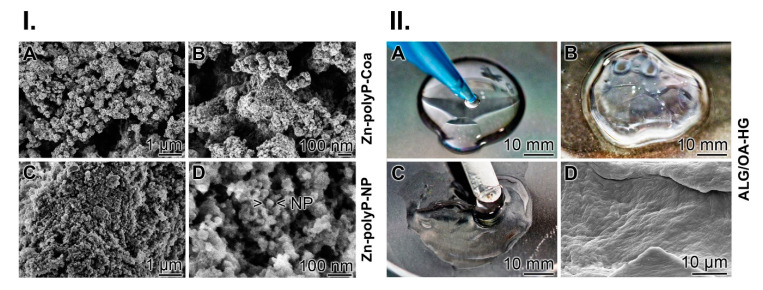
Formation of Zn-polyP coacervate and preparation of alginate-oxidized alginate hydrogel. (**I.**) Fabrication of Zn-polyP coacervate and Zn-polyP-NP; SEM. (**A**,**B**) Addition of ZnCl_2_ at super-stoichiometric concentration ratio to Na-polyP at pH 7 results in the formation of the aqueous coacervate phase which, after drying, appears as jagged material. (**C**,**D**) If the reaction is run at pH 10 nanoparticles (NP) are formed. (**II.**) Preparation of the alginate-oxidized alginate hydrogel. (**A**) Alginate was added dropwise to oxidized alginate (OA-50). (**B**) After gel formation (**C**) gelatin was added, resulting in an increased viscosity of the hydrogel. (**D**) Finally, the material was ionically cross-linked with ZnCl_2_ and freeze-dried; ESEM.

PolyP forms after exposure to metal cations, like Zn^2+^, either a coacervate or nanoparticles (NP) pH dependently. Addition of ZnCl_2_ to Na-polyP in a super-stoichiometric concentration ratio at pH 7 forms an aqueous Zn-polyP coacervate phase. The dispersed droplets and dense phase, after freeze-drying, appear as a jagged continuous lyophilic material that does not show discrete particles (Figure 2(I.A,B)). In contrast, if the reaction is performed at pH 10 solid globular particles are formed that comprise a diameter of ~30 nm (Figure 2(I.C,D)).

### 2.2. Preparation and Ionic Cross-Linking of the Hydrogel: ALG/OA-HG

At first sodium alginate was partially oxidized to the corresponding dialdehyde using sodium metaperiodate as the oxidizing agent. During this reaction, a specific oxidation of the free hydroxyl groups at C2 and C3 of the alginate monomeric units takes place, resulting in the formation of the corresponding dialdehydes with an ~50% degree of oxidation; OA-50 (Figure 2(II.A,B)). Then, this material was mixed with untreated alginate (2 parts of OA-50 and 3 parts of alginate) and cross-linked with gelatin in the presence of 0.1 M borax (Figure 2(II.C)). After ionic cross-linking with ZnCl_2_ and three subsequent washing cycles, the material was freeze-dried (Figure 2(II.D)); “ALG/OA-HG”.

### 2.3. Stability of the Ionic Cross-Linked Hydrogel: ALG/OA-HG

**Figure 3:**
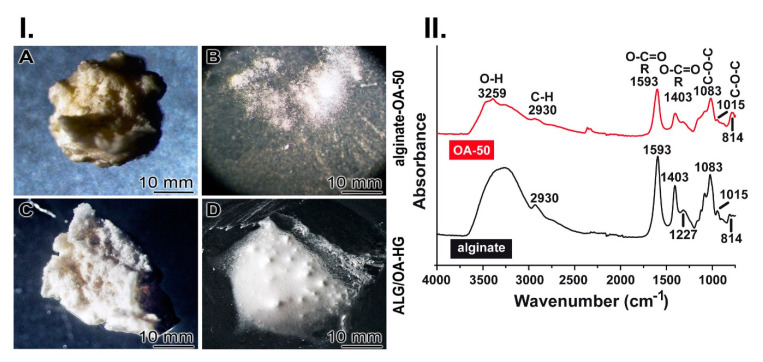
“ALG/OA-HG” and the characterization. (**I.**) Ionic cross-linking within the “ALG/OA-HG”. (**A**) Dry alginate-OA-50 material. (**B**) After submersion in saline the hybrid material dissolves almost completely after 12 h. (**C**) Solid alginate-OA-50 material obtained after treatment with ZnCl_2_ and drying. (***D***) After exposure to saline the material stays stable. (**II.**) Characterization of alginate and oxidized alginate (OA-50) by FTIR. Alginate shows the characteristic carboxylate (COO^−^) vibrational mode signals at 1593 cm^−1^ and 1403 cm^−1^. Then C-O-C stretching signals of the acetal group are recorded at 1083 cm^−1^ and at 1015 cm^−1^. The comparative FTIR spectrum to OA-50 shows that the signals for the latter material are less intensive.

During the process of cross-linking with ZnCl_2_ the material gains stability against dissolution in distilled water (Figure 3I.). If the dry alginate-OA-50 material (3 parts of alginate and 2 parts of OA-50) without gelatin (Figure 3(I.A)) is submersed for 12 h in saline an almost complete dissolution takes place (Figure 3(I.B)). However, if the alginate-OA material is treated with ZnCl_2_, then dried (Figure 3(I.C)) and subsequently exposed for 12 h to saline the hydrogel remains almost stable (Figure 3(I.D)); “ALG/OA-HG”.

### 2.4. Characterization of Oxidized Sodium Alginate by FTIR

The FTIR spectrum of Na-alginate shows a broad band at 3259 cm^−1^ indicating the presence of hydroxyl groups. The characteristic carboxylate (COO^−^) vibrational modes were observed as an anti-symmetric stretch at 1593 cm^−1^ and a symmetric stretch at 1403 cm^−1^. The bands corresponding to the symmetrical C-O-C stretching of the acetal group (1083 cm^−1^ and 814 cm^−1^) and the anti-symmetrical C-O-C stretch (1015 cm^−1^) are also present as described [12]; Figure 3II. Compared with the Na-alginate, the spectrum for OA-50 showed overall less intensive signals. In addition, the stretching vibrations for C-O-C detected at 1015 cm^−1^ were likewise significantly reduced as a result of the chain cleavages. These data indicate the transformation of alginate to OA-50 via periodate oxidation [13].

### 2.5. Addition of PolyP to the Hydrogel: ALG/OA-polyP-HG

**Figure 4:**
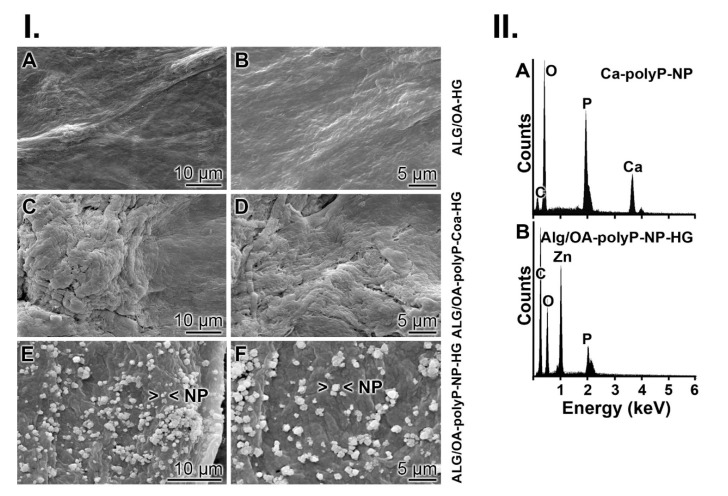
Morphology and characterization of the hydrogels. (**I.**) Surface texture of the different hydrogels; ESEM. (**A**,**B**) The polyP lacking hydrogel, “ALG/OA-HG”. This hydrogel was supplemented with Na-polyP and then treated with ZnCl_2_. (**C**,**D**) At pH 7 polyP forms a coacervate, “ALG/OA-polyP-Coa-HG”, while (**E**,**F**) the surface of the material, composed of alginate/OA-50/gelatin and polyP, and obtained at pH 10, is covered with nanoparticles (NP), “ALG/OA-polyP-NP-HG”. (**II.**) EDX spectra, taken from (**A**) Ca-polyP-NP and (**B**) “ALG/OA-polyP-NP-HG”.

The surface structure of the “ALG/OA-HG” hybrid material, after treatment with ZnCl_2_, is largely flat. Only some texture/structure is visible that originates from the random distribution and associations of the alginate-OA-50 blocks (Figure 4(I.A,B)). This freeze-dried alginate/oxidized alginate-based material, “ALG/OA-HG”, was supplemented with polyP.

Addition of Zn-polyP and subsequent incubation at pH 7 results in the formation of more structured but still plain surfaces (Figure 4(I.C,D)); “ALG/OA-polyP-Coa-HG” comprising polyP in the coacervate phase. If the reaction process is performed at pH 10, polyP forms with Zn^2+^ nanoparticles (Figure 4(I.E,F)); “ALG/OA-polyP-NP-HG”. Those particles have a dimension of 200 nm and are apparently aggregates from individual ~20 nm sized NP (Figure 2(I.D)).

Determination of the rheological properties of the alginate/oxidized alginate-based material with polyP at pH 7 revealed an increase in the rheological behavior from 0.21 ± 0.05 to 0.97 ± 0.12 Pa•s, after the addition of ZnCl_2_.

### 2.6. Presence of Zn^2+^ into “ALG/OA-polyP-NP-HG” Hybrid Material

The EDX technique was used to determine the presence in Zn^2+^ in the “ALG/OA-polyP-NP-HG” hybrid material. In comparison, the EDX spectrum for Ca-polyP nanoparticles (Ca-polyP-NP) was recorded. This pattern shows the characteristic signals for Ca and P, in addition to O and C (Figure 4(II.A)). If the spectrum was taken from the “ALG/OA-polyP-NP-HG” material the Zn signal shows up as 1.012 keV (L_α_ line) (Figure 4(II.B)). The atomic percentage was determined with 6.91% (correlated to the total weight).

### 2.7. Growth/Viability Dynamics of Keratinocytes on the Alginate/OA-50 Hydrogel

**Figure 5:**
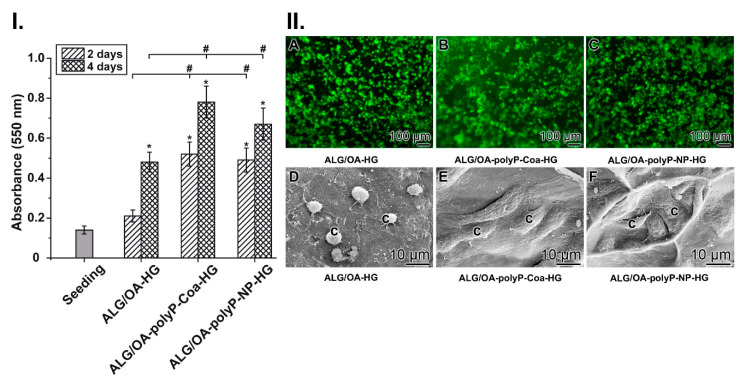
Viability of cells on the hydrogels. (**I.**) MTT analysis to assess the growth/viability of the keratinocytes. The cells were cultivated onto “ALG/OA-HG”, “ALG/OA-polyP-Coa-HG” or “ALG/OA-polyP-NP-HG” matrices. The viability/cell number was determined after an incubation period of 2 d or 4 d. Then the cells were reacted with MTT and the intensity of the formed formazan dye was measured with a microplate reader at 550 nm. Ten parallel assays per point were performed. The data represent means ± SD. The significances within an individual group (*; *p* < 0.01) were calculated. The significances with respect to the assays without polyP are likewise marked (#; *p* < 0.01). (**II.**) Microscopic analysis of the cells onto the respective gels, “ALG/OA-HG”, “ALG/OA-polyP-Coa-HG” or “ALG/OA-polyP-NP-HG”, after an incubation period for 4 d. In **A** to **C** the cells were vitally stained with calcein and inspected with a fluorescence microscope. In **D** to **F** the cells were visualized by ESEM.

The viability of the keratinocytes was recorded after an incubation for 2 or 4 days using the MTT reaction. At the beginning of the incubation the cultures (seeding) gave a formazan dye intensity of 0.14 ± 0.02 (absorbance units). In the “ALG/OA-HG” cultures, this value increased after an incubation period of 2 d to 0.21 ± 0.03 and after 4 d to 0.48 ± 0.05 (absorbance units, respectively). However, if the cells were cultivated onto the polyP-supplemented matrices the values, reflecting the number of cells, increased significantly by 2.5-fold (1.6-fold) for the “ALG/OA-polyP-Coa-HG” series and by 2.4-fold (1.4-fold) for the cultures onto “ALG/OA-polyP-NP-HG” after an incubation period for 2 d and 4 d, respectively (Figure 5I.).

The data obtained with the MTT assay were confirmed also by eye inspection of the cells (Figure 5(II.A–C)) and by ESEM observation (Figure 5(II.D–F)). The cells were incubated onto the matrices for 4 d. Staining the cells with calcein shows that the keratinocytes are less abundantly distributed on the “ALG/OA-HG”, in comparison to the presence of the cells on the polyP-containing matrices, “ALG/OA-polyP-Coa-HG” and “ALG/OA-polyP-NP-HG” (Figure 5(II.B,C) versus Figure 5(II.A)). ESEM inspection revealed a distinct difference in the morphology of the cells present on the polyP-free (Figure 5(II.D)) and the polyP-supplemented hydrogels (Figure 5(II.E,F)). While the cells on the “ALG/OA-HG” matrix are round and less abundant, the cells which were cultured on the polyP-containing matrices show a well spreading morphology.

### 2.8. Keratinocyte Migration Assay

**Figure 6:**
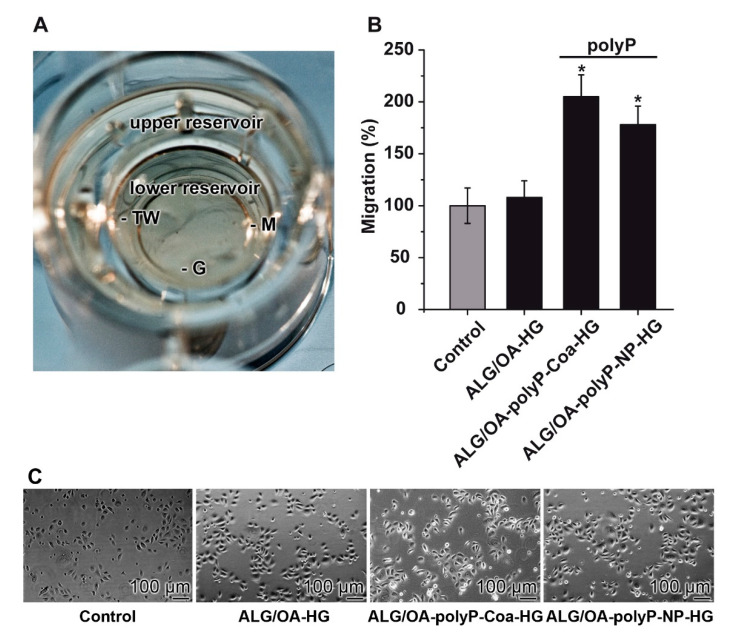
Determination of the migration activity of keratinocytes in the Boyden chamber. (**A**) Image of the Boyden chamber. The cells were placed into the upper reservoir, a transwell insert (TW), which is connected with the lower reservoir by a porous membrane (M). On the bottom of the lower reservoir, the hydrogel (G) is introduced. Attracted by the polyP released from the gel the cells migrate through the membrane. (**B**) Increase of the migration activity of keratinocytes in those assays into which the polyP-containing hydrogels, either “ALG/OA-polyP-Coa-HG” or “ALG/OA-polyP-NP-HG” had been added to the lower chamber. The migration activity is correlated with the one measured in the controls (without any hydrogel; set to 100%). Addition of the polyP-lacking “ALG/OA-HG” hydrogel to the system did not influence the activity (*; *p* < 0.01). (**C**) Light micrographs of the cells on the bottom side of the membrane (invasive cells) prior to fixation.

The Corning-HTS Transwell-24 [Boyden chamber] system was used for the quantification of the migration propensity of the keratinocytes (Figure 6A). The hydrogel was inserted into the lower reservoir of the chamber. Then the Transwell system with the upper reservoir was inserted and the migration activity of the keratinocytes was determined, as described under ‘‘Materials and Methods’’.

After an incubation period of 12 h the number of cells spreading on the bottom side were counted. The number of the controls (absence of any hydrogel in the system) was set to 100%. Addition of “ALG/OA-HG” to the lower chamber had only an insignificant influence on the migration activity. However, if the gels in the lower chamber were supplemented with polyP, “ALG/OA-polyP-Coa-HG” or “ALG/OA-polyP-NP-HG” a very significant increase in the migration activity of about 2-fold in the assays with “ALG/OA-polyP-Coa-HG” and of 1.7-fold in the system with “ALG/OA-polyP-NP-HG” is measured (Figure 6B). Prior to fixation the distribution of the cells on the bottom side of the Boyden membrane, after incubation with the different hydrogels and—in parallel—also in the control assay are shown (Figure 6C).

### 2.9. Effect of the Materials in the Short Term Skin Irritation Test

**Figure 7:**
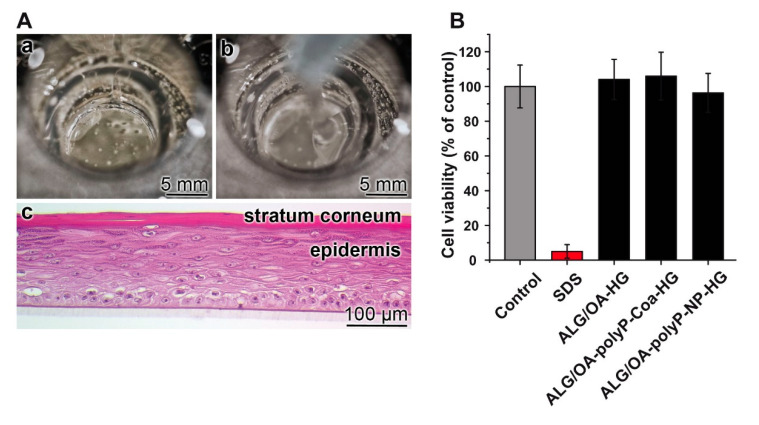
Determination of the viability of the hydrogels by using the in vitro reconstructed 3D human skin model. (**A**) the skin sample in the inserts of the well plates (**a**) prior and (**b**) after the addition of the hydrogel sample (200 µL). (**c**) Histological cross-section through the skin with the stratum corneum and the epidermis; staining with hematoxylin and eosin. (**B**) Short term skin irritation test by application of the MTT assay (*n* = 5).

The EpiDerm skin model EPI-200 was applied to assess the potential local inflammatory reaction as well as the eventual damage caused by the alginate-based wound-healing gel. The assay system contained in the inserts of the 24-well plates the reconstructed epidermis samples (Figure 7(Aa)). Then the hydrogel samples in a volume of 200 µL were overlayed (Figure 7(Ab)). In Figure 7(Ac) a histological cross-section is shown with the layers: stratum corneum and epidermis. For the determination of the effect on cell viability, the MTT assay was applied. PBS was used as a control (no additive) and 5% SDS (sodium dodecyl sulfate) as a strong irritation agent. With SDS the cell viability was reduced down to 5.3 ± 3.9%. The four different hydrogels did not affect the viability of the cells significantly (Figure 7B).

## 3. Discussion

PolyP is a physiological polymer that is synthesized in every metazoan cell and stored in special organelles, in lysosomes, dense granules of the acidocalcisomes, mitochondria, and nuclei [14,15,16]. Data indicate that the synthesis starts in the mitochondria through the formation ATP, which is released from this organelle and channeled through the envelope of the acidocalcisomes, a process during which polyP is synthesized (reviewed in: [8]). The characteristic feature of polyP is the property of the polymer to form a coacervate in the presence of divalent cations at neutral pH and at higher pH (around 10) nanoparticles [17]. The coacervate phase is the immediate physiologically active phase, while the solid nano-particulate form can be considered as a depot form which is transformed in medium at pH 7 to the aqueous coacervate phase. Our group has characterized this transformation with Ca-polyP [17], while the Zn-polyP coacervate has been introduced by Wang et al. [18].

Until this study, it has not been attempted to integrate polyP into an alginate/oxidized-alginate matrix, together with gelatin. The hydrogel was formed with oxidized-alginate having a 50% yield of metaperiodate-caused oxidation. This material readily forms with non-oxidized alginate a matrix, which is primarily linked by ionic interactions and allows binding of gelatin via Schiff base formation. Prior to Zn^2+^ exposure and the subsequent ionic linkage formation the “ALG/OA-HG” hydrogel is readily dissolvable. However, after cross-linking with Zn^2+^ the material becomes more resistant. The FTIR analyses showed that the alginate polymer is converted into oxidized-alginate as could be deduced from the overall reduction in the intensities of the signals reflecting the hydroxyl groups, as well as the characteristic carboxylate and acetal groups within the polymer. Finally, it was demonstrated by EDX analysis that the Zn element is present in the hydrogel with about 7%.

Since the hydrogel still exposes unoccupied ionically charged side groups an ionic interaction with polyP via Zn^2+^ was conveniently possible. Consequently, the biologically active component, polyP, was bound to the matrix in a stable fashion. This property makes the interaction of polyP with alginate/oxidized-alginate-gelatin superior in comparison to approaches based on binding of bone morphogenetic proteins or other cytokines to the alginate-based material. The latter interaction is basically only an electrostatically one [19,20]. Interestingly, it could be demonstrated by ESEM analysis that the Zn-polyP containing hydrogel, which was prepared at pH 7 to allow coacervate formation exposes a smooth surface. In contrast, when polyP was added to the alginate/oxidized-alginate-gelatin hydrogel, distinct Zn-polyP nanoparticles were formed that decorated the surface of the matrix. This result indicates that the material formed at pH 10 contains integrated nanoparticles, which consequently act as a depot for the polyP ingredient.

The adhesion process of cells to a given matrix is dependent upon a suitable physico-mechanical precondition of the substrate and the supply of energy required as a fuel for the adhesion energy demands for the interaction between the cells and the extracellular scaffold [21]. The first prerequisite is met by the gelatin constituent in the hydrogel, which provides the R-G-D signature for the cell membrane integrated integrins [22]. The energy demand is covered by extracellular polyP, which provides ATP to both the extracellular and intracellular compartments [8]. Since these two active components are present in the fabricated “ALG/OA-polyP-HG” matrix, both in “ALG/OA-polyP-NP-HG” and in “ALG/OA-polyP-Coa-HG”, it was expected and then experimentally proven that the polyP supplemented alginate matrices are excellent matrices for cells to adhere and also to grow on them. Already by eye-inspection it was apparent that the number of cells attaching on the “ALG/OA-HG” surface is considerably lower than that of the polyP matrices “ALG/OA-polyP-NP-HG” and “ALG/OA-polyP-Coa-HG”. The ESEM inspections disclosed that the cells associated with the polyP-free matrix are ball-like and do not show any spreading. The latter property is seen for all cells attached to the polyP-containing hydrogels; cell spreading is a sign for actively growing and differentiating cells [23]. The quantitative assessment of the growth rate/viability of the keratinocytes was documented by application of the MTT assay. The viability of the cells seeded onto polyP-containing alginate matrices is about 2-fold higher compared to the one measured for cells cultured onto polyP-free hydrogel.

Closely related to the effect of polyP present in the hydrogel on cell attachment and cell proliferation is the effect on cell migration. Cell migration is at least as energy consuming as cell adhesion [24]. Using the Boyden chamber assay, it is documented that the rate of cell migration in the polyP-containing hydrogels is substantially upregulated compared to the matrices that lack this polymer. Therefore, the material we described here is a gel-like material, similar to the one described by the group of Mooney [25]. Studies using an in vitro model based on reconstructed human epidermis gave no hints for an adverse, potential local inflammatory reaction caused by the polyP-based hydrogel materials and underscored their potential usefulness in wound healing.

## 4. Materials and Methods

### 4.1. Materials

Na-polyphosphate (Na-polyP) with an average chain length of 40 phosphate units was purchased from Chemische Fabrik Budenheim (Budenheim; Germany). Sodium alginate (alginic acid from the brown algae giant kelp *Macrocystis pyrifera*; 250 cps for 2% at 25 °C; #W201502) with a M/G molar ratio 60%/38% according to ^1^H-NMR analysis [26], sodium metaperiodate (#S1878), ZnCl_2_ (#208086), CaCl_2_ (#1.72580 SAFC), ethylene glycol (anhydrous, 99.8%; #324558) and gelatin from porcine skin (#G2500) were from Sigma-Aldrich (Taufkirchen; Germany).

### 4.2. Preparation of Zn-polyP-coacervate and Zn-polyP-nanoparticles

In one series of experiments, the coacervate and nanoparticle phases of Zn-polyP were fabricated by using a different pH during preparation [27]. In brief, Na-polyP in aqueous solution (2 g in 100 mL) was adjusted to pH 7 (with 200 mM Tris-HCl; #RES3098T-B7, Sigma) and then supplemented, dropwise, with 5.4 g of ZnCl_2_ (in 50 mL of water), followed by stirring overnight. Then the gel-like precipitate was washed with cold distilled water, once with ethanol and then dried at 60 °C for 24 h; ‘‘Zn-polyP-Coa’’. The nanoparticles (NP) were prepared similarly, but the reaction was run at pH 10; ‘‘Zn-polyP-NP’’.

For comparative studies, Ca-polyP nanoparticles (Ca-polyP-NP) were prepared as described before [28].

### 4.3. Preparation of Alginate Dialdehyde

The strategy for the preparation of alginate dialdehyde described by Jejurikar et al. [13] was applied. A solution of Na-alginate (8.0 g, 40.4 mmol uronate) was dispersed in 100 mL ethanol. Then, sodium metaperiodate (0.25 M) was added to the alginate/ethanol suspension and stirred in the dark at room temperature for 24 h, followed by quenching with equimolar amounts of ethylene glycol. The degree of oxidation was adjusted by varying the concentration of the metaperiodate. Usually, a periodate equivalent of 50% results in a yield of oxidation of 48% [29]. In order to remove non-reacted metaperiodate the material was dissolved in 50% ethanol and washed three times with this solvent. During this procedure the color changed from yellowish to white. The final material was freed of periodate (silver nitrate proof [13]). Finally, the material was freeze-dried. Applying this procedure, the periodate oxidation procedure occurs randomly, but initially, only one carbohydrate unit in a chain is oxidized before the next oxidative attack on the chain occurs. During this process, a reduction of the size of the chains occurs [30]. Usually, an oxidation yield of ~50% for alginate was used for the studies; “OA-50”.

### 4.4. Fabrication of Hydrogels

The hydrogels were prepared by utilizing both covalent and ionic cross-linking potencies of OA-50 [13,31]. OA-50 was covalently linked by using the aldehyde groups that are formed after cleavage of the C2-C3 bond within the uronic acid subunits. These groups can spontaneously react with adjacent hydroxyl groups on other uronic acid subunits in the polymer chain under formation of cyclic intramolecular or intermolecular hemiacetals, resulting in a reduction of the number of aldehyde groups [13,32,33]. The covalent linkage formation is followed by immersion and ionic cross-linking with divalent cations. During this procedure the gels shrink by ~20%. Following this rational 1 mL of a 3% (*w*/*v*) alginate solution, prepared in 0.1 M borax (pH 9.4; #221732 Sigma-Aldrich) and a 2% (*w*/*v*) OA-50 solution was mixed with 2 mL of gelatin (2 mg/mL) to obtain the hybrid hydrogels allowing reversible Schiff base reaction. Then the gel was supplemented with 0.5 mL ZnCl_2_ (2 mg/mL), washed and freeze-dried; alginate/oxidized alginate hydrogel “ALG/OA-HG”.

### 4.5. Rheological Measurements

The determinations were carried out in an analogue rotational viscometer PCE-RVI-1 (PCE Instruments, Southampton, UK) at a rotational speed of 12 rpm, at the dimensions of 300 × 300 × 450 mm. The results are given in Pa•s.

### 4.6. Addition of PolyP to the Hydrogel

The hybrid hydrogel (10 mL), prepared from alginate, oxidized alginate and gelatin and cross-linked with Zn^2+^, was mixed with 5 mL Na-polyP (0.5 mg/mL) and then ionically cross-linked, with 0.5 mL ZnCl_2_ (20 mg/mL in distilled water; #793523 Sigma) at two different pH conditions, at pH 7.0 and at pH 10.0 (adjusted with NaOH). After an incubation for 1 h the material was washed in distilled water and freeze-dried; ALG/OA-polyP-HG. Due to a recent study at pH 7 a Zn-polyP coacervate is formed, while at pH 10 Zn-polyP is organized as nanoparticles within the hydrogel; “ALG/OA-polyP-Coa-HG” and “ALG/OA-polyP-NP-HG”.

### 4.7. Fourier Transformed Infrared Spectroscopy

The material was ground to a micro-milled powder and analyzed in an ATR (attenuated total reflectance)-FTIR spectroscope/Varian 660-IR spectrometer (Agilent, Santa Clara, CA, USA) for Fourier transformed infrared spectroscopy (FTIR).

### 4.8. Energy-Dispersive X-ray Spectroscopy

The energy-dispersive X-ray spectroscopy (EDX) analyses were performed with an EDAX Genesis EDX System included to a scanning electron microscope (Nova 600 Nanolab, FEI, Eindhoven; The Netherlands).

### 4.9. Cultivation of Human Epidermal Keratinocytes

The commercially available adult human epidermal keratinocytes were obtained (#102-05A, Sigma-Aldrich). They were cultivated in human epidermal keratinocyte complete culture medium (#SCMK001, Sigma-Aldrich) as described [34]. The cells were incubated in 24-well plates at 37 °C. Prior to addition, the wells were coated with 200 µL of the indicated hydrogel sample. The hydrogels were cross-linked in the wells with ZnCl_2_ to allow a 1.5 mm thick layer to form. Then, the cultures were started with 2 × 10^4^ cells per well in a total volume of 1 mL. After an incubation period of 96 h the cell viability was determined using the XTT assay.

In the second series of experiments, the cells were inspected by ESEM.after critical point drying (at 43 °C) after transfer into 2% [*v*/*v*] aqueous glutaraldehyde, osmium oxide treatment and processing through the acetone dehydration series [35].

### 4.10. Cell Viability/MTT Assay

Growth/viability of the keratinocytes was quantitated using the MTT (3-[4,5-methylthiazol-2-yl]-2,5-diphenyl-tetrazolium bromide (# M6494, Thermo Fisher Scientific, Dreieich, Germany) assay [36]. In short, keratinocytes (2 × 10^4^ cells/mL) were seeded in 24-well plates, coated with the indicated hydrogel, and incubated for 2 or 4 days. Then the assays were supplemented with 50 μL of MTT (10 mg/mL), followed by the addition of the solubilization buffer (10% SDS with 0.01 N HCl). After overnight incubation the intensity of the developed color was determined at 550 nm in a microplate reader. Ten parallel assays per point were performed.

Parallel samples were taken and vitally stained with calcein (#17783, Sigma). The fluorescent label was recorded with the fluorescence microscope—or in a second approach by ESEM after critical point drying (at 43 °C) after transfer into 2% [*v*/*v*] aqueous glutaraldehyde, osmium oxide treatment, and processing through the acetone dehydration series [35].

### 4.11. Keratinocyte Migration Assay

The Corning-HTS Transwell-24 [Boyden chamber] (pore size, 3 µm; #CLS3398; Sigma-Aldrich) was used to quantify migration of the keratinocytes, basically as described [26,37] and using the cells in the human epidermal keratinocyte complete culture medium. The bottom chambers of Transwell were coated with 100 µL of the respective hydrogel sample and then cross-linked with ZnCl_2_. After 30 min, the gel formed was exhaustedly washed with medium. The cell suspensions (2 × 10^4^ cells per well in 1 mL) were added to the upper chambers (the inserts) of the Boyden system. After an incubation period of 12 h cells spreading on the bottom side of the membrane (invasive cells) were fixed with cold 4% formaldehyde, then washed in PBS (phosphate-buffered saline), stained with 10 µM propidium iodide (Invitrogen, Karlsruhe, Germany) and counted. The experiments were performed in quadruplicate and repeated at least 3 times.

### 4.12. In Vitro Skin Irritation Assay

The EpiDerm Skin Irritation Test (EPI-200) purchased from MatTek (Ashland, MA, USA) was used in this study as a model system of human epidermis [38,39]. The 24-well plates of this in vitro skin irritation test contained in the inserts the reconstructed epidermis samples. These tissue samples developed from normal human-derived epidermal keratinocytes and differentiated to the basal, spinous [stratum spinosum] and also the granular layers. Finally, the tissue was overlayed with a multilayered stratum corneum. The cells were covered with 200 µL of the respective hydrogel (“ALG/OA-HG” hydrogel, as well as the polyP containing gels “ALG/OA-polyP-Coa-HG”, and “ALG/OA-polyP-NP-HG”) and incubated for 120 min. PBS was used as a control (no additive); 5% SDS (sodium dodecyl sulfate) reduced the cell viability (MTT assay) to 5.3 ± 3.9%. The artificial skin was sectioned and then stained with hematoxylin/eosin [40].

### 4.13. Microscopic Analyses

Electron microscopy was performed with an environmental scanning electron microscope (ESEM) using an ESEM XL-30 apparatus (Philips, Eindhoven, Netherlands). Scanning electron microscope (SEM) images were taken with a HITACHI SU8000 electron microscope (Hitachi, Krefeld, Germany). Light microscopical images were taken with a VHX-600 Digital Microscope from Keyence (Neu-Isenburg, Germany).

The fluorescent label was visualized with the fluorescence microscope (Olympus, Hamburg, Germany) using the wavelengths 496 nm (excitation) and 520 nm (emission).

### 4.14. Statistical Analysis

After verification that the respective values follow a standard normal Gaussian distribution and that the variances of the respective groups are equal, the results were statistically assessed using the independent two-sample Student’s *t*-test [41].

## 5. Future Directions

In a recent study, we outlined the beneficial effects of a Zn^2+^ supplement in a material to be applied for wound healing [27]. This material was biocompatible and significantly stimulated growth of keratinocytes in vitro. Future studies are planned to investigate the potency of the Zn-polyP-based hydrogels to induce ATP synthesis in keratinocytes, as is already known for human umbilical vein endothelial cells [42].

**Figure 8:**
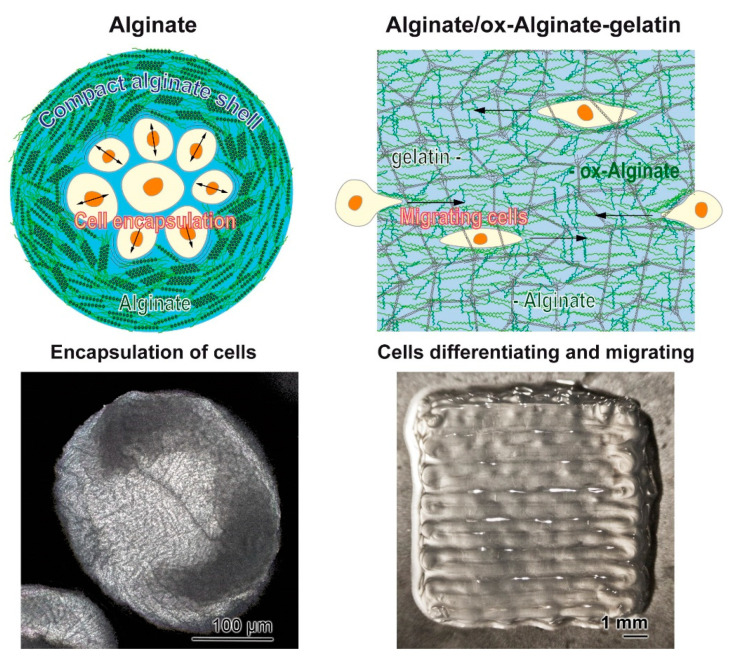
Schematic outline of alginate spheres (**left**) and alginate/oxidized-alginate-gelatin hybrid hydrogel (**right**). (**Left**) The natural alginate comprises a compact hydrogel that is ionically linked to a resistant protection of cells against external attacks. (**Right**) The alginate/oxidized-alginate-collagen hybrid material is a relatively soft hydrogel into which cells can migrate and proliferate. This material is designed as wound dressing.

In a previous study, we could establish that polyP, added in a nano-particulate form, significantly accelerates wound healing in vivo in normal mice, and in particular, in diabetic mice [43]. In the present contribution, we provide a suitable gel-like hydrogel that can be straightforwardly used as a gel or paste, which should also be efficiently active in vivo as a protecting and regeneration-inducing wound dressing. Addition of oxidized-alginate transforms the relatively resistant alginate hydrogel to a soft hybrid material, composed of a relatively rigid alginate scaffold around porous structures that are formed and stabilized by an oxidized-alginate-gelatin hydrogel into which polyP is embedded and ionically cross-linked via Zn^2+^ (Figure 8). Even more, the presented gel offers a new avenue to induce regeneration in wound healing, not only in a morphogenetic way, but also by supporting the migration of keratinocytes to the damaged area and providing a dynamic environment for the newly formed tissue to undergo physiological wound contraction [44].
